# Pigeonpea Hybrid-Proline-Rich Protein (CcHyPRP) Confers Biotic and Abiotic Stress Tolerance in Transgenic Rice

**DOI:** 10.3389/fpls.2015.01167

**Published:** 2016-01-22

**Authors:** Sunitha Mellacheruvu, Srinath Tamirisa, Dashavantha Reddy Vudem, Venkateswara Rao Khareedu

**Affiliations:** Centre for Plant Molecular Biology, Osmania UniversityHyderabad, India

**Keywords:** abiotic stress, blast disease, endochitinase, hybrid proline rich protein, pigeonpea, transgenic rice

## Abstract

In this study, we report the overexpression of *Cajanus cajan* hybrid-proline-rich protein encoding gene (*CcHyPRP*) in rice which resulted in increased tolerance to both abiotic and biotic stresses. Compared to the control plants, the transgenic rice lines, expressing *Cc*HyPRP, exhibited high-level tolerance against major abiotic stresses, viz., drought, salinity, and heat, as evidenced by increased biomass, chlorophyll content, survival rate, root, and shoot growth. Further, transgenic rice lines showed increased panicle size and grain number compared to the control plants under different stress conditions. The *CcHyPRP* transgenics, as compared to the control, revealed enhanced activities of catalase and superoxide dismutase (SOD) enzymes and reduced malondialdehyde (MDA) levels. Expression pattern of *Cc*HyPRP::GFP fusion-protein confirmed its predominant localization in cell walls. Moreover, the *CcHyPRP* transgenics, as compared to the control, exhibited increased resistance to the fungal pathogen *Magnaporthe grisea* which causes blast disease in rice. Higher levels of bZIP and endochitinase transcripts as well as endochitinase activity were observed in transgenic rice compared to the control plants. The overall results demonstrate the intrinsic role of *Cc*HyPRP in conferring multiple stress tolerance at the whole-plant level. The multipotent *CcHyPRP* seems promising as a prime candidate gene to fortify crop plants for enhanced tolerance/resistance to different stress factors.

## Introduction

Growth and development of living organisms are determined by diverse interactions between their genomes and environmental factors (Kohli et al., 2013). Plants have evolved complex signaling pathways—comprising of receptors, secondary messengers, phytohormones, and signal transducers—to sense various stresses and to acclimatize to different environmental conditions. These innate mechanisms facilitate transduction of stress signals for activation of stress-responsive gene expression to maintain plant growth and productivity (Yokotani et al., 2013). Multiple abiotic stresses proved to be potentially harmful and plants respond distinctly when compared with individual stresses (Atkinson and Urwin, 2012). The cellular and molecular responses of different plants to various environmental stresses have been studied extensively (Xiong et al., 2002). Different stresses, such as cold, heat, salt, and drought, trigger specific responses in plants with the help of specific sensors to generate adaptive mechanisms. Different families of TFs and their interacting cis-acting elements, known as regulons, have been characterized to play a key role in abiotic stress responses (Liu et al., 2014).

Abiotic stresses are known to be multigenic involving different metabolic pathways and hence difficult to engineer as compared to biotic stress factors (Vinocur and Altman, 2005).

The abiotic and biotic stress signaling pathways in plants share some common components (Walley and Dehesh, 2010). These components have revealed some convergent nodes of abiotic and biotic stress response pathways, and plants can rapidly adapt to a changed environment by synergistically or antagonistically regulating the signaling cross talk through these nodes (Xiao et al., 2013). In barley and tomato, osmotic and drought stresses resulted in enhanced resistance to fungal pathogens (Wiese et al., 2004; Achuo et al., 2006). RNAi suppression of *OsMPK5* gene and its kinase activity induced by ABA contributed to enhanced abiotic and biotic stress tolerance in rice (Sharma et al., 2013). Hence, it is necessary to identify regulators that connect both biotic and abiotic stress response pathways which provide opportunities for developing multiple stress tolerant crop plants.

Rice is the staple food for more than 3 billion people and is grown in different regions of the world. Rice plant is highly susceptible to drought stress especially at the reproductive stage, which results in a significant reduction in the grain yield (Price and Courtois, 1999). Molecular genetic studies have uncovered a number of regulatory genes which have been used for engineering stress tolerant rice varieties. Overexpression of rice ZFP182 protein conferred enhanced tolerance to rice plants against salt, cold and drought stresses (Huang et al., 2012). Transgenic rice plants expressing *Os*DIL were found to be more tolerant to drought stress during vegetative and reproductive stages (Guo et al., 2013a). Accordingly, there is a need to identify new genes and promoters for engineering food crops which can survive under different stress conditions with consistent yields.

Plant hybrid-proline-rich proteins (HyPRPs) are putative cell-wall proteins consisting of a repetitive proline-rich (PR) N-terminal domain and a conserved eight-cysteine-motif (8 CM) C-terminal domain (Dvoráková et al., 2011). Though they are ubiquitous in plants, yet little is known about their roles other than as cell-wall structural proteins (Yeom et al., 2012). Proteins containing 8 CM motif have different functions in storage, protection, enzyme inhibition, lipid transfer, and cell-wall structure (Jose-Estanyol et al., 2004). Further, based on the presence of a proline-rich domain and a secretary signal, HyPRPs have been designated as a group of secreted structural cell-wall proline-rich proteins (Priyanka et al., 2010). *HyPRP* genes are usually expressed in a tissue-specific manner or induced by specific stresses or hormones but their role in development, as well as in biotic and abiotic stress tolerance are not clearly understood (Jose-Estanyol et al., 2004). *MsPRP2* is a proline-rich protein encoding gene of *Medicago sativa* which was induced by water-deficit conditions (Deutch and Winicov, 1995). A hybrid-proline-rich protein encoding gene (*CcHyPRP*) of *Cajanus cajan* was strongly induced by salt, drought, heat, and ABA treatments (Priyanka et al., 2010). The *Arabidopsis* HyPRP gene EARLY ARABIDOPSIS ALUMINUM INDUCED 1(*EARLI1)* was induced during seed germination and was strongly expressed in certain parts of the seedlings (Xu et al., 2011). In soybean, the expression profile of *SbPRP* gene was modulated by ABA, internal circadian rhythm, as well as abiotic and biotic stress factors (He et al., 2002).

In the present study, *CcHyPRP* of *C. cajan* was introduced into rice and transgenic plants were evaluated against different stresses. Transgenic rice plants expressing *Cc*HyPRP exhibited increased levels of tolerance to drought, salt, and heat stresses at different stages of plant growth and development. Furthermore, *CcHyPR*P-transgenic rice lines showed enhanced levels of resistance against the leaf blast disease caused by *Magnaporthe grisea*.

## Materials and methods

### Construction of *CcHyPRP*-overexpression vector for rice transformation

The *CcHyPRP*-expression units, driven by either constitutive CaMV 35S and/or inducible rd29A promoter along with *nos* terminator, were excised with *Hind*III restriction enzyme from pBI121 vector, and were cloned independently in pSB11CaMV35S*bar* vector (Supplemental Figure 1A). The recombinant clones, pSB11CaMV35S*bar*-CaMV35S*CcHyPRP*, and pSB11CaMV35S*bar*-rd29A*CcHyPRP*, were maintained in HB101 cells and mobilized independently into *A. tumefaciens* strain LBA4404 by triparental mating (Ramesh et al., 2004). The resulting superbinary vectors were designated as pSB111CaMV35S*bar*-CaMV35S*CcHyPRP* and pSB111CaMV35S*bar*-rd29A*CcHyPRP*.

### *Agrobacterium*-mediated stable transformation of rice

Seeds of the popular indica rice cultivar, BPT5204, were used for genetic transformation experiments. Callus derived from the scutellum of mature embryo was infected with the *Agrobacterium* culture induced with PIM II medium supplemented with 100 μM acetosyringone as described (Ramesh et al., 2004). Putatively transformed calli, after 4 weeks of incubation on the MS medium containing 6.0–8.0 mg L^−1^ phosphinothricin (PPT), were selected and cultured on the proliferation medium for 2 weeks. Later, the actively growing calli were transferred onto the regeneration medium containing BAP (3–4 mg L^−1^) and NAA (0.1–0.5 mg L^−1^) (Ramesh et al., 2004). Regenerated shoots were transferred onto the 1/2 MS rooting medium, and rooted plants were transferred to the pots and grown to maturity in the glasshouse. Putative transgenic plants along with control plants were tested for their tolerance to the herbicide BASTA as described (Nagadhara et al., 2003).

### Molecular analysis of transgenic plants

Genomic DNA was isolated from the BASTA-tolerant transgenics and control plants as per the method of McCouch et al. (1988). PCR analysis was carried out using *CcHyPRP* gene-specific forward 5′-ATGGCTTCCAAGGCTGCACTCCTC-3′ and reverse 5′-TTAAGCGCA GATGAAATCCTTAGG-3′ primers. Plasmid DNA was used as positive control, and the amplified products were separated on 1% agarose gel.

Southern blot analysis was done using 20 μg of genomic DNA digested with *Hind*III restriction enzyme. Digested DNA was separated on 0.8% agarose gel, and transferred to the N^+^ nylon membrane. Later, it was fixed by exposing to UV (1200 μJ for 60 s) in an UV cross linker (Sambrook and Russell, 2001). *CcHyPRP* (400 bp) coding region was used as a probe after labeling with AlkPhos Direct Labeling System (GE Healthcare). Pre-hybridization, hybridization and washing membrane were done according to the manufacturer's instructions.

Total RNA was isolated from 4-week-old control, CaMV35S*CcHyPRP*- and rd29A *CcHyPRP*- transgenic plants. The rd29A*CcHyPRP*-transgenic plants were subjected to 200 mM mannitol stress for 3 days prior to RNA isolation. Northern blot was carried out using 12 μg of total RNA. The separation of RNA on denaturing agarose gel, its transfer to nylon membrane and fixing were performed according to Sambrook and Russell (2001). Probe preparation, pre-hybridization, hybridization, and washing steps were carried out according to the manufacturer's instructions.

### Localization of *CcHyPRP-GFP* fusion-protein in stable transformed cells of rice

The coding sequence of *CcHyPRP* was fused upstream to the green fluorescent protein (GFP) coding region. The fusion gene of *CcHyPRP-GFP-nos* was cloned in pCAMBIA3300-*bar* vector under the control of CaMV 35S promoter. Plasmid vector containing CaMV35S-*gfp*-*nos* was used as a positive control. Mobilization of the plasmid into the Agrobacterium and stable genetic transformation was done as described previously. Transformed cells of calli were observed under laser scanning confocal microscope (Leica Microsystems, Germany). Plasmolysis and protoplasts isolation from suspension cultures was carried out as described by Lee et al. (1989). Observations were recorded under the fluorescent microscope (Olympus BX41).

### Stress treatments and assay of enzyme activities

Two-week-old control and transgenic plants were subjected to NaCl (250 mM) and mannitol (250 mM), independently, for 3 days. For heat stress, plants were exposed to 48°C for 2 h, and were allowed to recover for 3 days under normal conditions at 30°C.

### Determination of catalase activity

Leaf samples collected from the stressed plants were homogenized in 50 mM phosphate buffer (pH 7.0). The homogenate was centrifuged at 8000 g for 20 min at 4°C. Enzyme extract was added to hydrogen peroxide-phosphate buffer (pH 7.0), and the time required for the decrease in the absorbance at 240 nm from 0.45 to 0.40 was noted. Enzyme solution containing hydrogen peroxide-free phosphate buffer was used as control. Catalse activity was determined according to Shin et al. (2012). The protein estimation was done using the Bradford reagent.

### Malondialdehyde (MDA) estimation

Leaf tissues were homogenized in 5 ml of 0.1% trichloroacetic acid (TCA). The homogenate was centrifuged at 5000 g for 10 min. The supernatant was collected and 500 μl of it was added to 4 ml of 20% TCA containing thiobarbituric acid (TBA) (0.5%). The mixture was heated at 95°C for 30 min, quickly cooled on ice, centrifuged at 5000 g for 15 min, and the absorbance of supernatant was read at 532 and 600 nm. After subtracting the non-specific absorbance at 600 nm, MDA concentration was calculated using an extinction coefficient of 155 mM^−1^ cm^−1^.

### Superoxide dismutase (SOD) activity

The SOD activity was assayed by monitoring the inhibition of photochemical reduction of nitroblue tetrazolium (NBT). The enzyme activity was estimated by adding 100 μl of enzyme extract to 3 ml of reaction mixture containing 50 mM potassium phosphate buffer (pH 7.8), 13 mM methionine, 75 μM NBT, 2 μM riboflavin, and 0.1 mM EDTA. The reaction mixture was illuminated at light intensity of 5000 lux for 15 min. The absorbance of samples was measured at 560 nm using spectrophotometer. One unit of SOD activity was defined as the amount of enzyme required to cause 50% inhibition of the reduction of NBT.

### Estimation of chlorophyll content

Leaf discs from 6-week-old transgenic and control plants were floated in 20 ml solution of NaCl (250 mM)/mannitol (250 mM) or water (experimental control) for 72 h at room temperature (28°C). For heat stress, leaf discs were floated in 20 ml of water kept at 42°C for 72 h. The leaf discs were ground using liquid nitrogen and dissolved in 80% acetone. Supernatant was collected after centrifuging the sample at 12,000 rpm for 15 min. Spectrophotometer readings taken at 663 and 645 nm were used for calculating the chlorophyll content.

### Evaluation of transgenic plants for various physiological and yield parameters

#### Seed germination assays

Four independent T_4_homozygous lines for each CaMV35S*CcHyPRP* (CR1, CR2, CR3, and CR4) and rd29A*CcHyPR*P (RR1, RR2, RR3, and RR4) constructs were selected for evaluation against different abiotic stresses. For germination assays, seeds were germinated on 0.5X MS medium (control) and the medium supplemented with mannitol (250 mM) or NaCl (250 mM). For heat stress, seeds were kept on 0.5X MS medium at 30°C for overnight followed by transferring them to 48°C for 2 h, and later transferred to normal (30°C) conditions. A seed was considered germinated when plumule and radicle are grown into a seedling. Seed germination rate (%) is calculated using the following formula: Number of seeds germinated/Total number of seeds tested × 100. Data on germination frequencies were recorded after 10 days of stress treatments.

#### Stress treatments at the seedling stage

To test the tolerance ability of plants during the initial stage of growth, 15-day-old seedlings were transferred to the Hoagland solution supplemented with mannitol (250 mM) or NaCl (250 mM) and allowed them to grow for 7 days. For heat stress treatment, plants were transferred to the incubator set at 48°C for 2 h, and moved to normal conditions. After 7 days of stress treatments, data on biomass, survival rate, root length, and shoot length were recorded and photographed. All the stress experiments were repeated at least three times.

#### Stress treatments at the vegetative stage

For analyzing the stress tolerance ability of transgenic plants at the vegetative stage (60–65 days old), drought (withholding water), and salinity (250 mM NaCl) stresses were applied for 15 days at 30°C. Later, the treated plants were allowed to grow under normal conditions. Heat stress treatments were given to the plants by subjecting them to 38°C for 2 h, 40°C for 2 h, 48°C for 2 h, and 52°C for 2 h. Later, plants were transferred to normal temperature (30°C) for the rest of the day. This cycle of treatments was repeated for 7 days. After different stress treatments, plants were allowed to grow to maturity under normal conditions (30°C). Data on panicle length, grain number and number of filled grains per panicle were recorded. In each treatment, 10 plants were used and all the experiments were repeated thrice.

#### Stress treatments at reproductive stage

To test the drought and salt tolerance abilities of transgenic plants at the reproductive stage (90–100 days old), drought (withholding water) and salt (250 mM NaCl) stress treatments were applied for 10 days at 30°C. After treatments, plants were transferred to normal conditions and allowed to grow to maturity. Heat stress treatments were given to the plants at 38°C for 2 h, 40°C for 2 h, and 42°C for 2 h followed by normal temperature (30°C) for the rest of the day. This cycle of treatments was repeated for 3 days. Data on panicle length and grain numbers per panicle were recorded. In each treatment, 10 plants were used and all the experiments were repeated thrice.

#### Evaluation of transgenic plants for *M. grisea* disease resistance

Seeds of CaMV35S-*CcHyPRP* transformants along with the seeds of untransformed controls were germinated in seedling beds. Fifteen-day-old seedlings were subjected to *M. grisea* strain IC9 (International Race C, Group 9, origin DRR, Hyderabad) infection using fully infected HR12 susceptible rice leaves. Disease symptoms were recorded after 15 days of infection and photographs were taken. Data on mean number of lesions and length of lesions as well as disease index (total leaf length affected/total leaf size × 100) were recorded. Resistance of transgenic plants was scored based on a scale of 0–9 as per Standard Evaluation System (SES) of International Rice Research Institute (1996).

#### Estimation of endochitinase activity

Leaf tissue (15 mg) was ground in the assay buffer consisting of 1 ml 10% of SDS, 1 ml of 10% Triton X-100, 2 ml of sodium EDTA (0.5 M), 70 μl of mercaptoethanol (14.4 M), and 96 ml of sodium acetate buffer (100 mM, pH 5.0). The amount of protein in the leaf sample was determined using the Bradford protein assay. Activity of the endochitinase present in samples was measured according to the manufacturer's instructions (Sigma Aldrich Chemical Co.). Fluorescence was determined at 360/460 (excitation/emission) with a spectrofluorometer (Jasco FP-600).

#### Real time PCR analysis

First strand cDNA was synthesized from RNA samples of control and transgenic seedlings subjected to blast stress for 15 days/150 mM mannitol treatment for 24 h along with unstressed plants. RT-PCR analysis was carried out using SYBR green master mix with Applied Biosystems 7500 real time PCR system at 94°C (1 min), 60°C (1 min), and 72°C (1 min) for 30 cycles. Later, products were analyzed through the melt curve analysis to check the specificity of PCR amplification. Each reaction was performed thrice, and the relative expression ratio was calculated using 2^−ΔΔct^ method employing actin gene as a reference (Tamirisa et al., 2014a). The genes for qRT-PCR were selected based on the predicted functional partners analyses with *Cc*HyPRP employing the STRING database (http://string-db.org). Since the *Cajanus cajan* is not on the string database, we have used the *Arabidopsis* information to find out the interactions.

#### Statistical analysis

Mean values, standard error and *t*-test were computed with the help of pre-loaded software in Excel, programmed for statistical calculations.

## Results

### Generation of *CcHyPRP*-transgenic rice lines

Employing the *Agrobacterium*-mediated genetic transformation method, a total of 20 independent transgenic rice lines of *CcHyPRP*, under the control of CaMV35S and rd29A promoters, were obtained. Out of them, four lines from each construct, viz., CR1, CR2, CR3, and CR4 (CaMV35S-*CcHyPRP*) and RR1, RR2, RR3, and RR4 (rd29A-*CcHyPRP*), were selected for functional analysis studies.

### Molecular analyses of transgenic rice lines

PCR analysis—using the DNA samples of Basta-tolerant plants employing *CcHyPRP* gene specific primers—revealed the presence of ~400 bp product representing the coding region of *CcHyPRP* gene, while no such band was observed in the control plants (data not shown). Southern blot analysis was carried out to confirm the presence of *CcHyPRP* expression unit in the genome of transgenic rice plants. Genomic DNAs (20 μg), isolated from the Basta- tolerant and PCR confirmed transgenics along with control plants, were used for Southern blot analysis. DNA digested with *Hind*III restriction enzyme, when probed with *CcHyPRP* coding region, revealed ~1.2 and ~1.4 kb bands representing *CcHyPRP* expression units driven by CaMV35S and rd29A promoters, respectively; whereas, no similar band was noticed in the untransformed control plants (Supplemental Figure 1B). Northern blot analysis, performed using the RNA isolated from the Southern-positive plants, disclosed varied intensities of a hybridizable band of >400 bp in different transgenic lines (Supplemental Figure 1C).

### Localization of *CcHyPRP*::GFP fusion-protein in stably transformed cells

**Figure 1:**
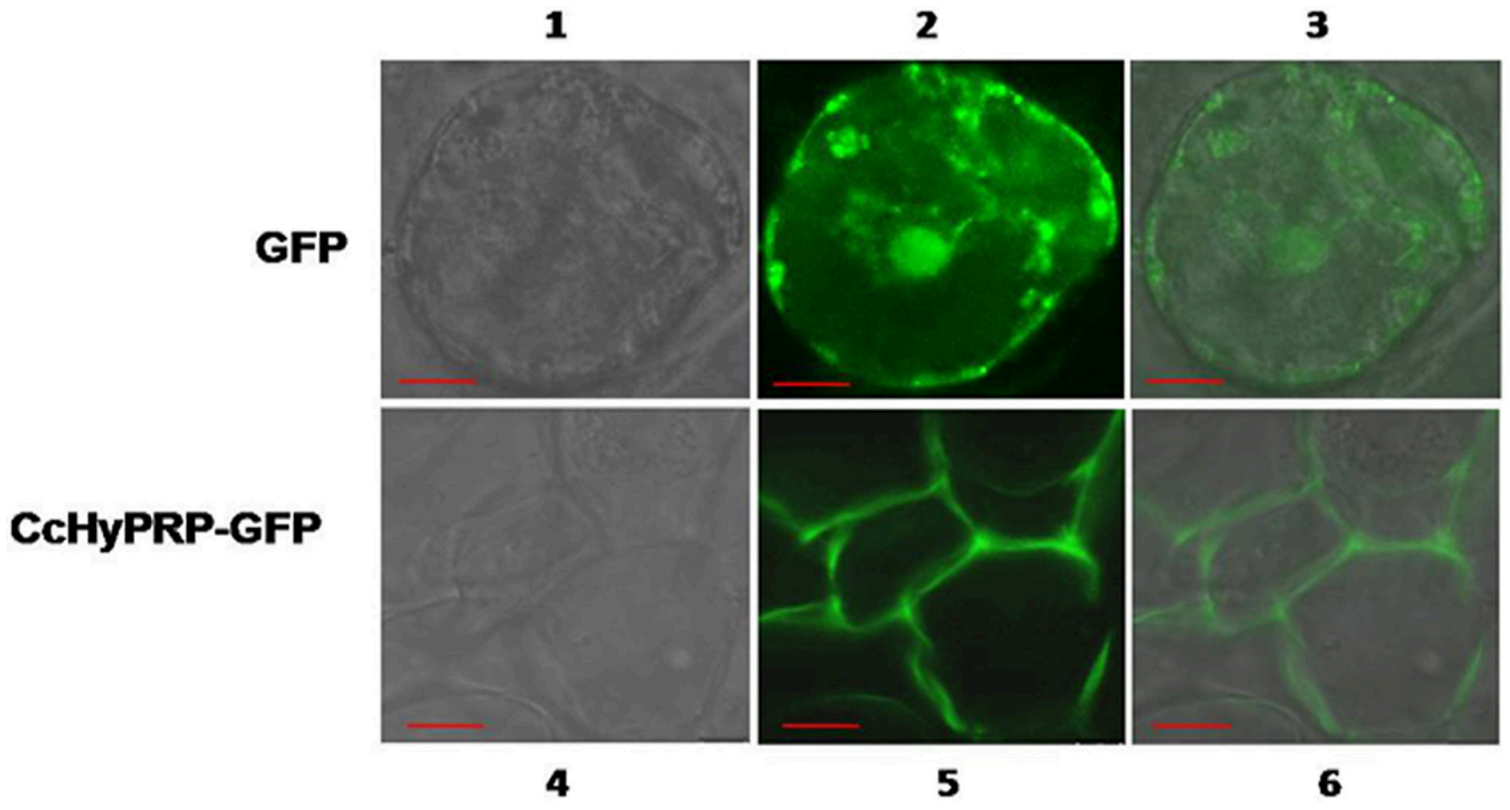
**Subcellular localization of *Cc*HyPRP protein in stably transformed cells of rice**. CaMV35S-*GFP* and CaMV35S-*CcHyPR*P***::****GFP* constructs were introduced into rice callus cells independently by *Agrobacterium*-mediated transformation. Individual cells derived from the transformed callus were observed under confocal microscope. Images 1, 4 are bright field and 2, 5 are dark field and 3, 6 combined. − = 100 μm.

Subcellular localization of the *Cc*HyPRP::GFP fusion-protein and GFP alone were examined in the stably transformed callus-derived cells of rice. An examination of the green fluorescence by laser-scanning confocal microscope showed fluorescence in the nucleus and cytoplasm of the cells expressing GFP alone, while cells expressing the fusion-protein exhibited fluorescence predominantly in the cell walls (Figure 1). No fluorescence was detected in the protoplasts of cells expressing the fusion-protein (Supplemental Figure 2A), while fluorescence was localized to the cell walls of plasmolysed cells (Supplemental Figure 2B).

### Tolerance levels of transgenics at different developmental stages against drought, salt, and heat stresses

#### Seed germination assay

**Figure 2:**
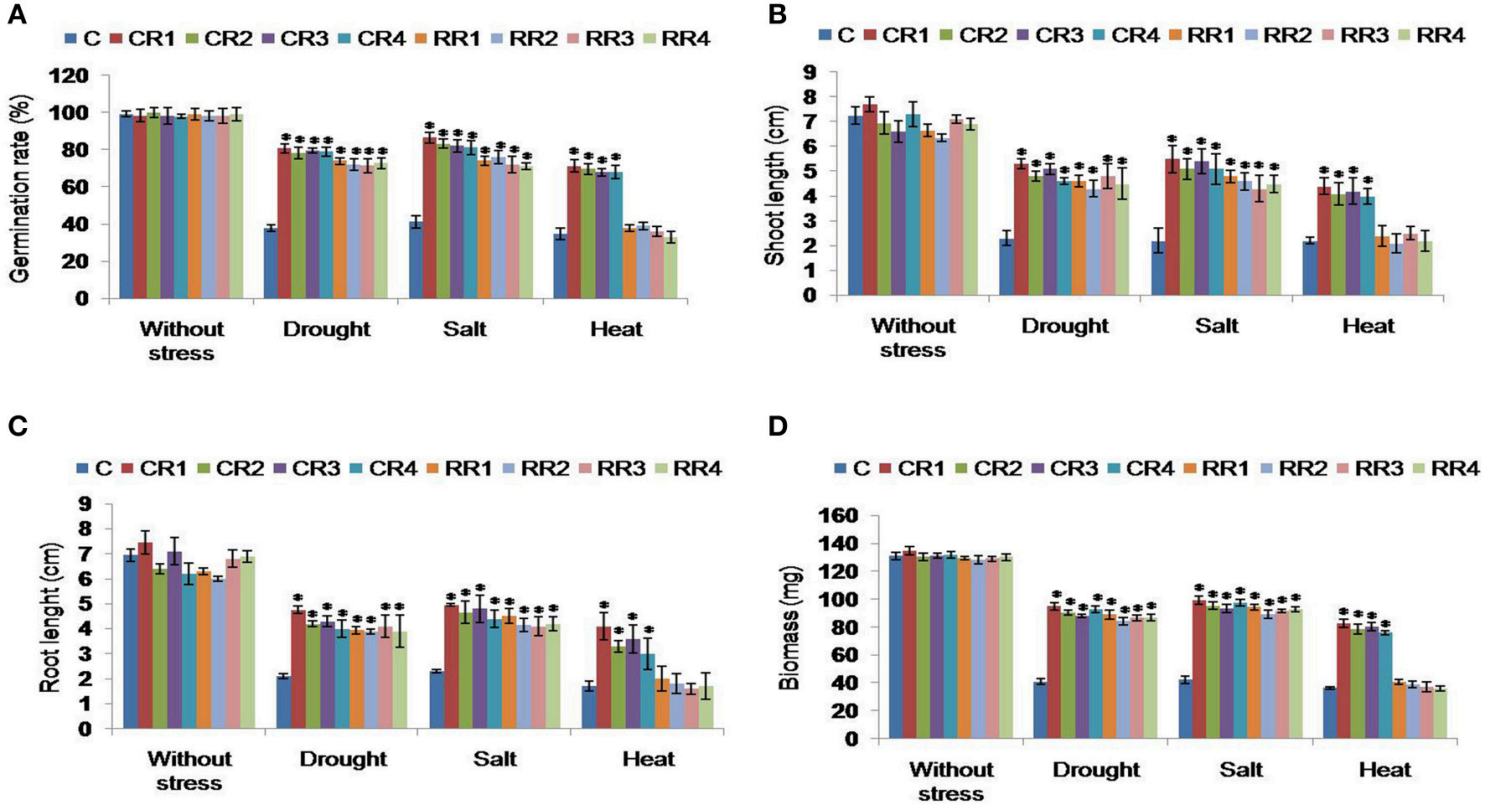
**Evaluation of *CcHyPRP*-transgenic lines against different abiotic stress conditions. (A)** Seed germination ability of control and transgenic plants was tested on MS medium supplemented with mannitol (250 mM), NaCl (250 mM) and heat (48°C for 2 h). Fifteen days old seedlings were transferred to the Hoagland solution supplemented with mannitol (250 mM) or NaCl (250 mM) and were allowed to grow for 7 days. For heat stress treatment, plants were transferred to the incubator set at 48°C for 2 h, and moved to normal (30°C) temperature. After 7 days of stress treatments, data on **(B)** shoot length, **(C)** root length, and **(D)** biomass were recorded and photographed. In each treatment, 10 seedlings of WT and transgenic lines were used. Bar represents mean and I represents SE from three independent experiments. ^*^indicates significant differences in comparison with the C at *P* < 0.05. WS represents without stress; CR1, CR2, CR3 and CR4 represent 35S transgenic lines; RR1, RR2, RR3, and RR4 represent rd29A transgenic lines; C represents control plants; FW represents fresh weight.

Under normal conditions, no differences were observed in the seed germination rates of transgenics and control plants. However, under stressed conditions, the transgenics showed higher seed germination rates as compared to the controls. Transgenic seeds subjected to mannitol and salt stress exhibited higher germination rates than that of control seed. Under heat stress, CR-transgenics revealed higher seed germination when compared to RR-transgenics and control plants (Figure 2A).

#### Effect of stress at the seedling stage

Transgenic seedlings (15 days old) subjected to mannitol and NaCl stresses showed higher survival rate, biomass, as well as increased shoot and root lengths as compared to the control plants (Figure 2; Supplemental Figures 3, 4). The shoot lengths of CR- and RR-transgenics under mannitol stress and salt stress were higher compared to the control plants (Figure 2B). However, under heat stress the CR-transgenics showed better response compared to RR-transgenics and control plants (Figure 2B). Similarly, the root lengths of CR- and RR-transgenics, under mannitol and salt stresses were higher compared to the control plants (Figure 2C). The CR-transgenics showed better growth response to heat stress when compared to the RR and control seedlings (Figure 2C). The biomass of CR- and RR-transgenics, under mannitol, and salt stresses were found to be higher than that of control plants (Figure 2D). However, under heat stress, the CR-transgenics exhibited increased biomass compared to the RR and control seedlings (Figure 2D).

#### Effect of stress at the vegetative stage

**Figure 3:**
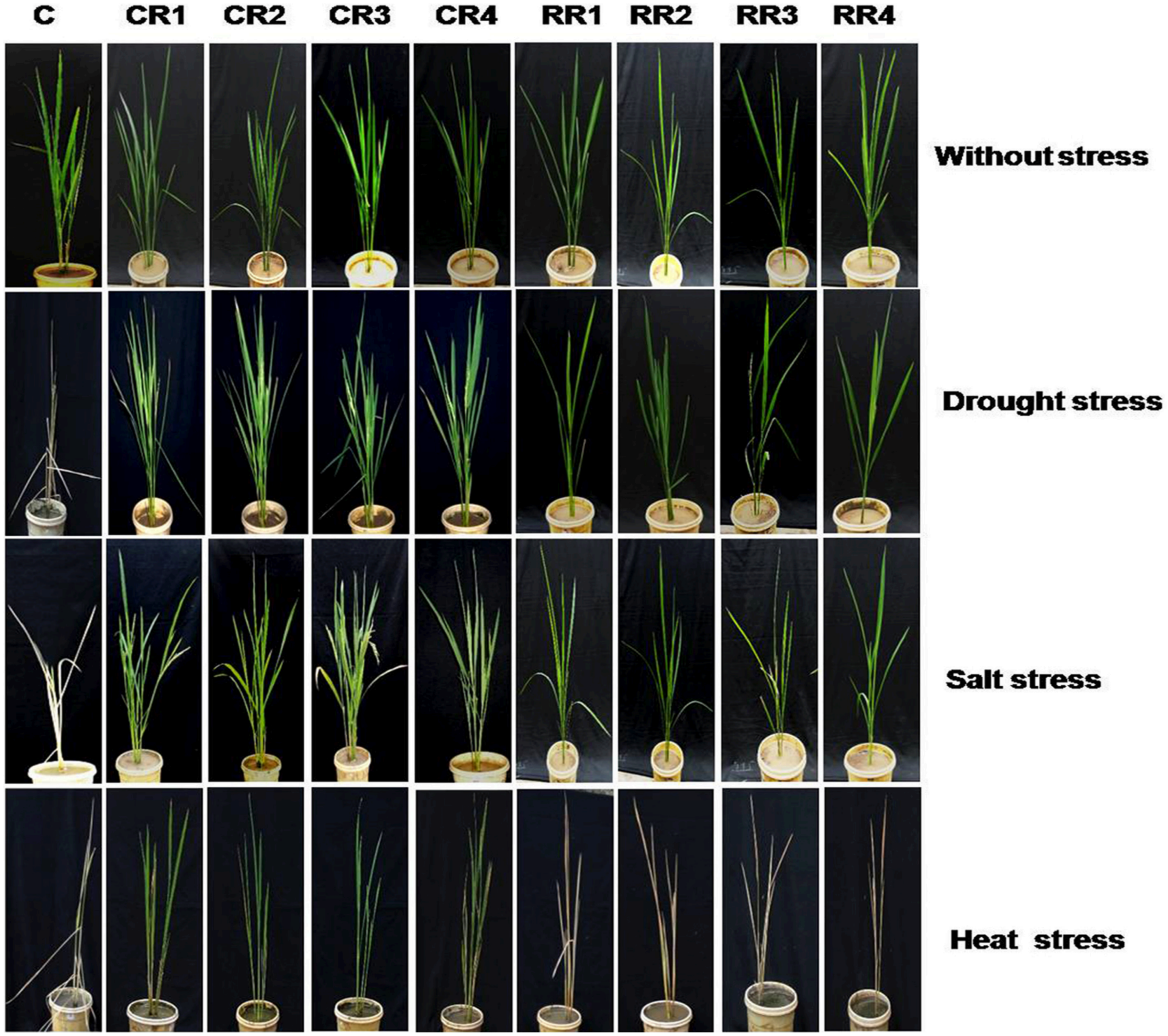
**Performance of *CcHyPRP*-transgenic plants against drought, salt and heat stress at the vegetative stage**. Control and transgenic plants (60–65 days old) were subjected to drought (withholding water) and salinity (250 mM NaCl) stresses for 15 days at 30°C. Later, the treated plants were allowed to grow under normal conditions. For heat stress treatments, plants were subjected to 38°C for 2 h, 40°C for 2 h, 48°C for 2 h, and 52°C for 2 h. Later, plants were transferred to normal temperature (30°C) for the rest of the day. This cycle of heat stress treatments was repeated for 7 days. Plants were allowed to grow under normal conditions for 20 days and later photographed. CR1, CR2, CR3, and CR4 represent 35S transgenic lines; RR1, RR2, RR3, and RR4 represent rd29A transgenic lines; C represents control plants.

Transgenic plants (60–65 days old), when subjected to drought and salt stresses, could reach the reproductive stage and set seeds as compared to the untransformed controls which failed to reach the reproductive stage (Figure 3).

#### Effect of stress at the reproductive stage

**Figure 4:**
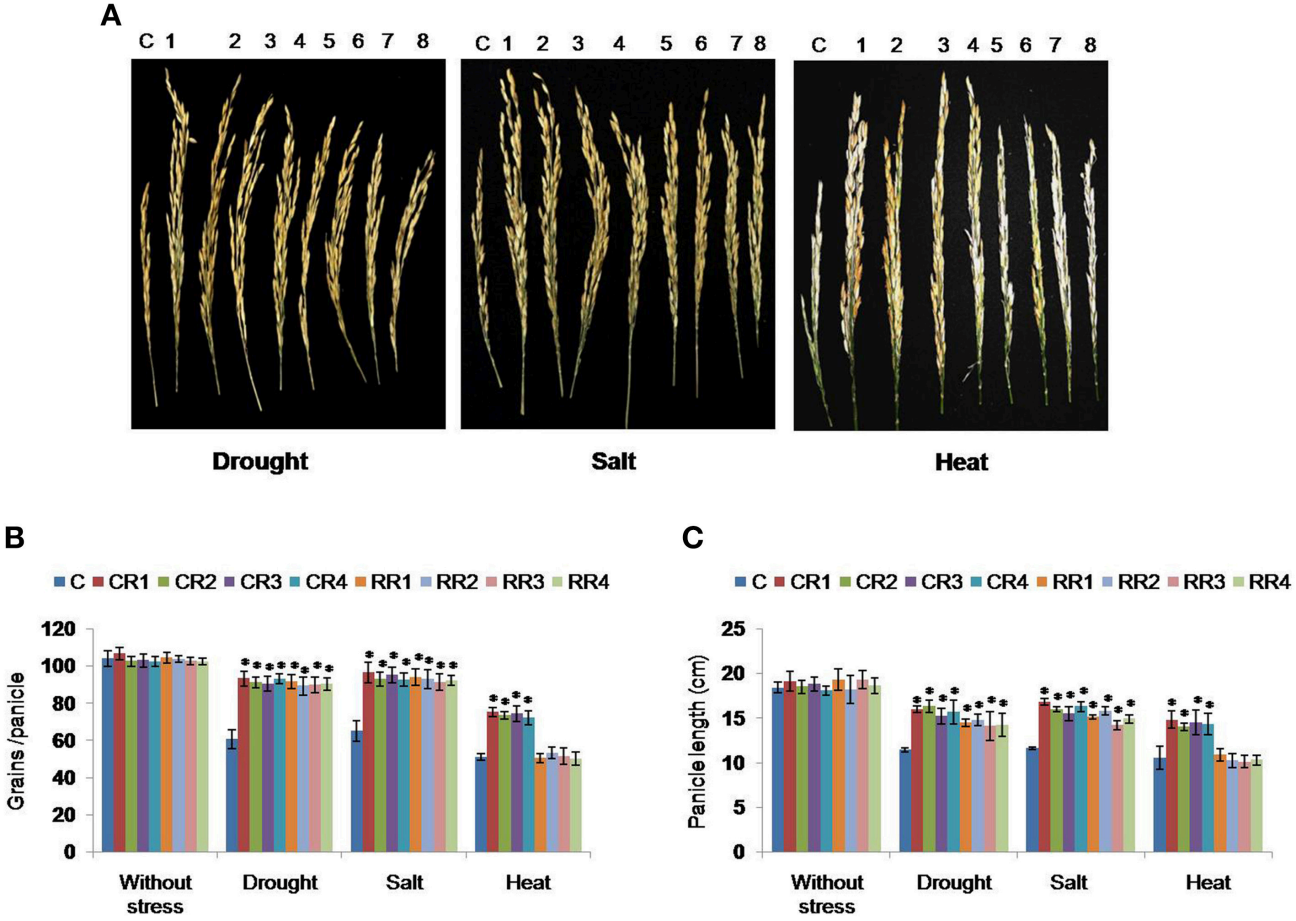
**Performance of *CcHyPRP*-transgenic plants against drought, salt and heat stress at the reproductive stage**. Transgenic and control (90–100 days old) plants were subjected to drought (withholding water) and salt (250 mM NaCl) stress treatments for 10 days at 30°C. Heat stress treatments were given at 38°C for 2 h, 40°C for 2 h, and 42°C for 2 h followed by transferring to normal temperature (30°C) for the rest of the day. This cycle of heat stress treatments was repeated for 3 days. **(A)** Panicle size and filled grains/panicle of control and transgenic rice plants subjected to stress treatments. Data on panicle length **(B)** and grain number/panicle **(C)** were recorded. In each treatment, 10 plants were used and all the experiments were repeated thrice. Bar represents mean and I represents SE from three independent experiments. ^*^indicates significant differences in comparison with the C at *P* < 0.05. 1, 2, 3, 4 represent CR-transgenics; 5, 6, 7, 8 represent RR-transgenics; CR1, CR2, CR3, and CR4 represent 35S transgenic lines; RR1, RR2, RR3, and RR4 represent rd29A transgenic lines; C represents control plants.

Transgenic plants of CR and RR at the reproductive stage (90–100 days old) showed longer panicles (Figure 4A) under drought (14.1 ± 1.65 and 16.3 ± 0.67 cm) and salt (16.8 ± 0.48 and 14.4 ± 0.37 cm) stresses when compared to the control (11.4 ± 0.2 cm) plants (Figure 4B). Moreover, transgenics also showed higher number of grains per panicle under drought (94.3 ± 3.92 and 89.3 ± 4.1) and salt (96.6 ± 5.6 and 91.3 ±4.3) stresses as compared to the control plants (62.6 ± 5.2) under similar stress conditions (Figure 4C). However, under heat stress conditions, CR-transgenics exhibited increased panicle length (14.5 ± 1.2 cm) and grain number (74.1 ± 3.8) compared to RR-transgenics and control plants. No significant differences were noticed between RR-transgenics and control plants under similar heat stress conditions (Figure 4).

#### Chlorophyll content in transgenic plants

**Figure 5:**
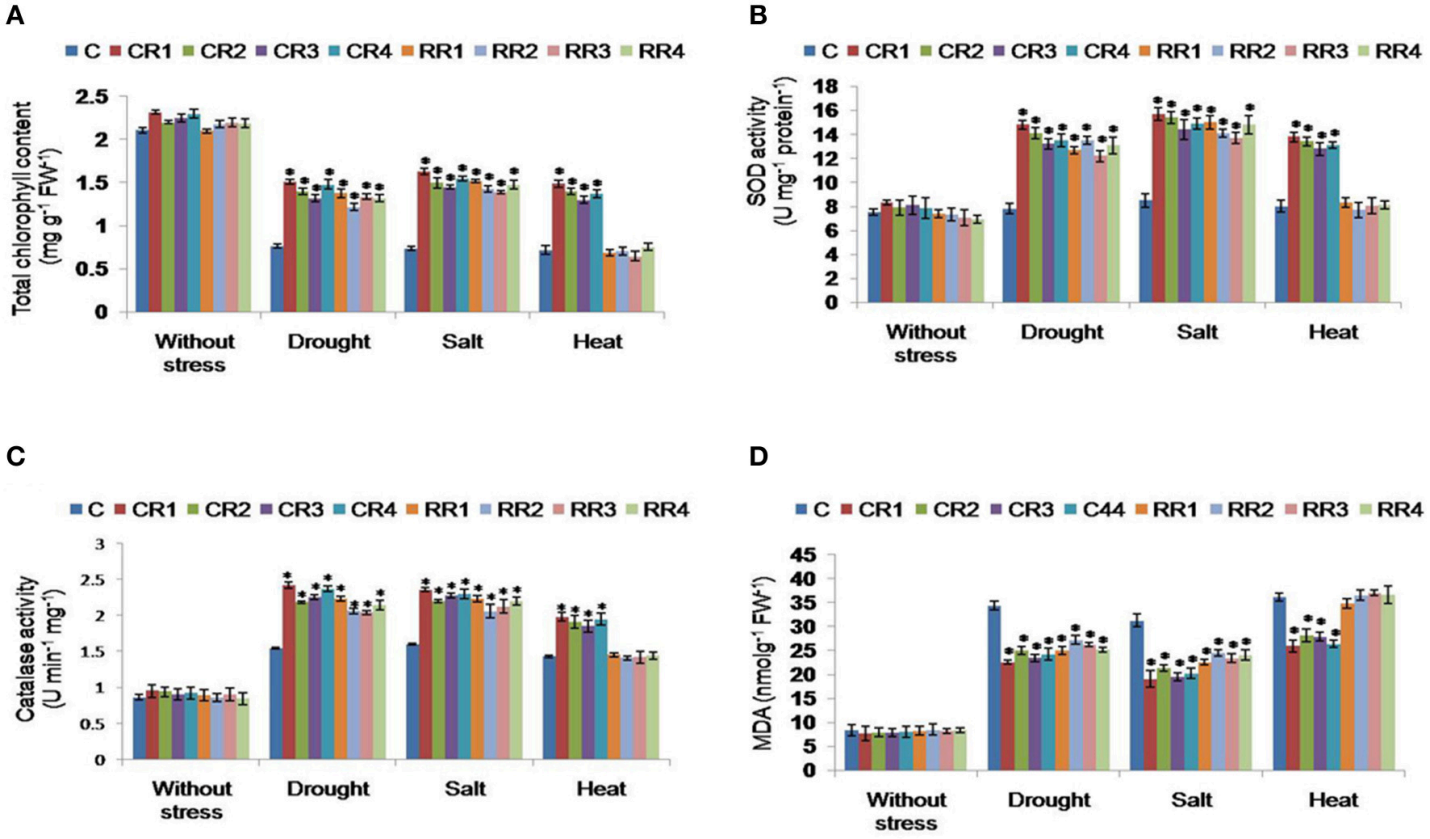
**Biochemical characterization of transgenic plants expressing *CcHyPRP* gene**. Two week old seedlings of control and transgenics were transferred to the Hoagland solution supplemented with mannitol (250 mM) or NaCl (250 mM) for 3 days and heat stress (48°C for 2 h) for estimation of Chlorophyll **(A)**, SOD **(B)**, Catalase **(C)**, and MDA **(D)**. Bar represents mean, and I represents SE from three independent experiments. For each treatment, 10 seedlings were used. ^*^indicates significant differences in comparison with the C at *P* < 0.05. C represents control plants; CR1, CR2, CR3, and CR4 represent 35S transgenic lines; RR1, RR2, RR3, and RR4 represent rd29A transgenic lines.

Leaf discs of CR- and RR-transgenic lines, subjected to mannitol and salt stresses, revealed higher chlorophyll contents compared to the control plants grown under similar conditions (Figure 5A). CR-transgenics, subjected to heat stress, showed higher levels of chlorophyll content compared to RR-transgenics and control plants (Figure 5A).

#### SOD activity in *CcHyPRP*-transgenic lines

The total SOD activity was significantly higher in *CcHyPRP*-transgenics, when compared to untransformed control plants, under drought, NaCl and heat stress conditions (Figure 5B). SOD activity was significantly increased in the transgenic plants subjected to mannitol and salt stress conditions when compared to the activity recorded in the control plants under similar stresses (Figure 5B). The CR-transgenics disclosed increased SOD activity under heat stress conditions. Whereas, under heat stress conditions no significant differences were observed between RR-transgenics and control plants. In transgenics and control plants, increased levels of SOD activities were observed under stressed conditions as compared to the normal stress-free conditions (Figure 5B).

#### Catalase activity in *CcHyPRP*-transgenic lines

Increased levels of catalase activity were observed in CR-transgenics under normal and heat stress conditions compared to RR-transgenics and control plants. Catalase activity was significantly increased in transgenic plants under mannitol and salt stresses compared to the activity recorded for control plants under similar stresses (Figure 5C). Under heat stress treatment, CR-transgenics showed higher catalase activity while there was no significant difference between RR-transgenics and control plants (Figure 5C).

#### MDA content in *CcHyPRP*-transgenic lines

MDA content was higher in control plants compared to that of CR and RR transgenics under mannitol and salt stress conditions. However, under heat stress conditions, MDA levels were significantly reduced in CR-transgenics as compared to RR and control plants. MDA content in transgenic plants subjected to mannitol and salt stresses were significantly lower as compared to the control plants (Figure 5D). Whereas under heat stress, CR transgenics showed lower MDA concentrations as compared to RR and control plants. Under unstressed normal conditions, there was no significant difference between the MDA concentrations of transgenic and control plants (Figure 5D).

#### Transgenic plants showing resistance to *M. grisea*

**Figure 6:**
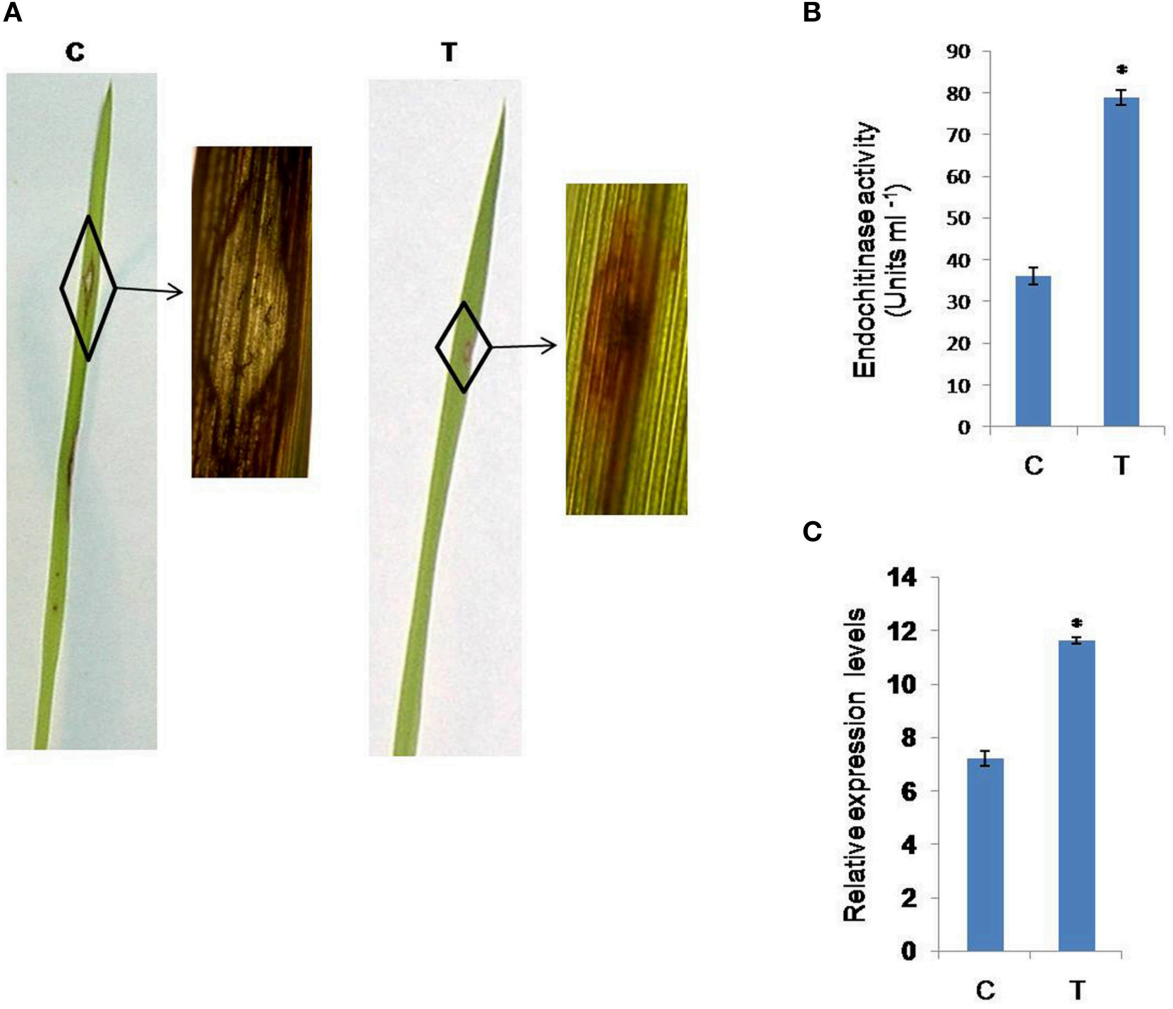
**Evaluation of *CcHyPRP*-transgenic rice against blast disease caused by *Magnaporthe grisea***. Blast disease reaction in control and transgenic plants after 15 days of *M. grisea* inoculation (infection). Appearance of disease symptoms on leaves of **(A)**: Untransformed control and *CcHyPRP*****-**transgenic rice **(B)** Estimation of endochitinase activity in transgenic lines (T) and control (C) plants. **(C)** Comparison of the relative transcript levels of endochitinase gene in transgenic (T) and control (C) plants. Actin was used as an internal control. The vertical column indicates the relative transcript level. Bar represents mean and I represents SE from three independent experiments. ^*^indicates significant differences in comparison with the C at *P* < 0.05. T represents 35S transgenic line; C represents untransformed control plant.

The CaMV35S-*CcHyPRP* plants exhibited greater resistance against *M. grisea*, compared to the control plants, as evidenced by the lesser number of leaf lesions and reduced lesion length (Figure 6A). Transgenic plants showed a mean number of 3.0 ± 0.7 lesions/leaf when compared to 9.3 ± 1.08 lesions/leaf observed in the control plants. Similarly, the transgenic plants disclosed a mean lesion length of 0.63 ± 0.10 mm compared to 1.8 ± 0.14 mm in the control plants. Transgenic plants showed reduced disease index (35.5%) compared to untransformed plants (62%). When compared to untransformed plants, transgenic plants exhibited a SES score of 3.0–5.0 on 0–9 scale.

#### Endochitinase activity in transgenic plants

The endochitinase activity was measured in *M. grisea* infected *CcHyPRP*-transgenics and untransformed control rice plants. A significantly higher level of endochitinase activity was observed in the transgenic plants as compared to the untransformed controls under similar stress conditions (Figure 6B).

#### Expression analysis of endochitinase gene in CaMV35 transgenic plants by qRT- PCR

RT PCR analysis was carried out to analyze the expression levels of endochitinase gene in *CcHyPRP*-transgenic plants infected with *M. grisea*. The results revealed substantial increases in the relative expression levels of endochitinase gene under the biotic stress conditions. However, the expression levels of the gene were significantly lower in untransformed control plants under similar stress conditions (Figure 6C).

#### Expression analysis of bZIP, LTP, and ATPase genes in *CcHyPRP*-transgenic plants by qRT- PCR

**Figure 7:**
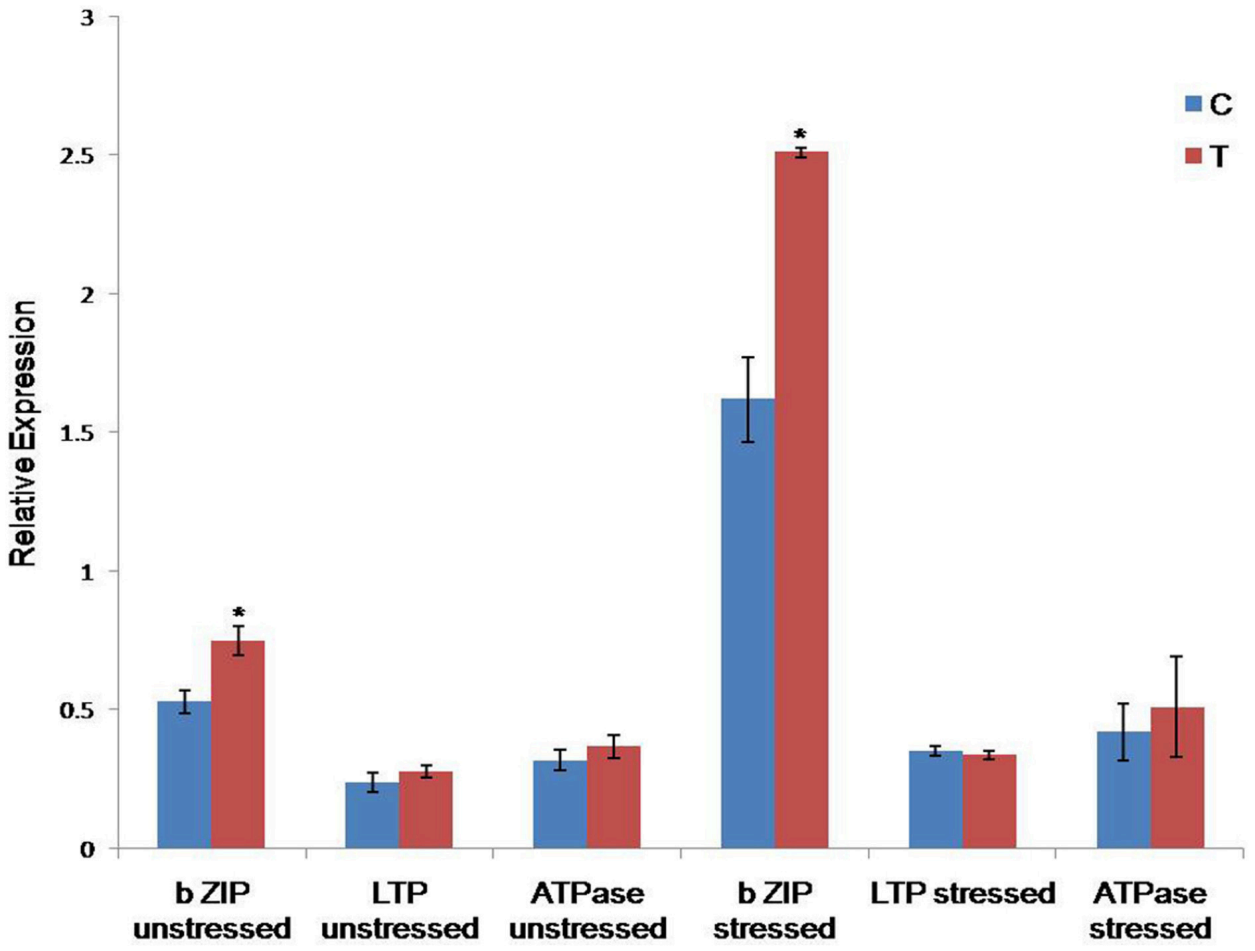
**Analysis of expression profiles of bZIP, LTP and ATPase genes by real-time PCR under normal and drought stress conditions**. Comparison of the relative transcript levels of bZIP, LTP, and ATPase in control (C) and transgenic (T) plants subjected to unstressed and 150 mM mannitol stress for 24 h. Actin was used as an internal control. The vertical column indicates the relative transcript level. Bar represents mean and I represents SE from three independent experiments. ^*^indicates significant differences in comparison with the C at *P* < 0.05.

RT PCR analysis was carried out to analyze the expression profiles of three selected genes, viz., bZIP transcription factor (LOC_Os08g43090), lipid transfer protein (LOC_Os03g01320), and vacuolar ATPase (LOC_Os02g34510**)** under stressed and unstressed conditions. These genes were selected based on the predicted functional partners analyses with *Cc*HyPRP employing the STRING database (http://string-db.org). Transgenic plants showed higher expression levels of bZIP transcription factor compared to the control plants under stress and unstressed conditions (Figure 7). However, the expression levels of LTP and ATPase genes were not significantly different from that of control plants under similar conditions.

## Discussion

Different biological processes are affected when the living organisms are exposed to diverse climatic and other environmental stresses. Plants being sessile are bestowed with distinct mechanisms to withstand and adapt to both biotic and abiotic stresses. Occurrence of different abiotic stresses is the major challenge encountered by plants and they have developed suitable adaptive mechanisms to perceive and transmit stress signals to the cellular compartments (Duan and Cai, 2012). Abiotic stresses such as extreme temperatures and water deficit conditions often constrain the growth and productivity of major crop species including cereals (Barnabás et al., 2008). As such, there is an urgent need for engineering the cereal crops for heat and drought tolerance since they are sensitive and vulnerable to these stresses, especially, at the reproductive stage which is responsible for low crop productivity. Pigeonpea *Cc*HyPRP contains a highly conserved hydrophobic 8 CM domain in the C-terminal half which is a key feature of the hybrid proline-rich proteins (HyPRPs). Genes encoding HyPRPs are expressed in a tissue-specific manner and are induced by various stresses and hormones. The same 8 CM domain, which has hydrophilic character, is found in the lipid transfer proteins, and these proteins are known to play a vital role in both biotic and abiotic stresses (Garcia-Olmedo et al., 1995; Jose-Estanyol et al., 2004). Transgenic *Arabidopsis* plants, expressing the *CcHyPRP* gene, exhibited an increased tolerance toward multiple abiotic stresses such as drought, salt and heat stresses (Priyanka et al., 2010).

To test the functionality of *CcHyPRP* in crop plants, the cointegrated super-binary vector pSB111-*bar- CcHyPRP* has been used to transform rice by employing the protocols optimized in our laboratory (Nagadhara et al., 2003; Ramesh et al., 2004). The presence of ~1.2/1.4 kb hybridizable band with *CcHyPRP* probe in different transformants indicates the integration of the intact expression unit of *CcHyPRP* in the rice genome (Supplemental Figure 1). Localization studies revealed the presence of *Cc*HyPRP::GFP fusion-protein predominantly in the cell walls of stably transformed rice cells (Figure 1). Furthermore, the absence of fluorescence in the isolated protoplast amply suggests that *Cc*HyPRP is a cell wall associated protein (Supplemental Figure 2).

Transgenic rice plants expressing *CcHyPRP* gene, under the control of either constitutive (CaMV35S) or stress-inducible (rd29A) promoter, were evaluated against drought, salt and heat stresses. Transgenic lines of CR1, CR2, CR3, and CR4, when tested against drought, salt and heat stresses, revealed marked tolerance as compared to the control plants. Whereas, RR1, RR2, RR3, and RR4 transformants exhibited tolerance to drought and salt stresses but were found to be sensitive to the heat stress. Apparently, the susceptibility of RR transgenics to heat stress is attributable to the absence of heat-shock-inducible elements in the rd29A promoter (Priyanka et al., 2010).

Under drought, salt and heat stress conditions, *CcHyPRP* transgenic plants disclosed higher seed germination rate, survival rate, increased root, and shoot growth besides enhanced plant biomass when compared to the control plants (Figure 2; Supplemental Figures 3, 4). Transgenic rice plants expressing *Cc*HyPRP could grow to maturity and set seeds while the control plants failed to survive under similar stress conditions (Figure 3). These results amply suggest that *CcHyPRP* gene plays a remarkable role in regulating the abiotic stress tolerance. Ectopic expression of *Pennisetum glaucum* vacuolar Na+/H+ antiporter in rice resulted in higher seed germination rates as compared to the wild type plants (Verma et al., 2007). Transgenic rice and *Arabidopsis* expressing *Os*LEA3-2 and *Cc*CDR showed superior growth performance than that of wild type plants under salinity and osmotic stress conditions (Duan and Cai, 2012; Tamirisa et al., 2014a).

Transgenic *CcHyPRP*-plants disclosed increased panicle size and higher number of filled grains when compared to the control plants under drought, salt and heat stress conditions (Figure 4). The yield potential of cereals is mainly dependent on the sensitivity to stress at the reproductive and grain-filling stages (Barnabás et al., 2008). Water-deficit during reproductive stage, especially at meiosis, reduced the seed set by 35–75% in various cultivars of bread wheat (Saini and Aspinall, 1981) and rice (Sheoran and Saini, 1996). Heat-stress related yield loss in cereals is due to the high temperature-induced shortening of developmental phases and crop duration (Stone, 2001), besides adverse effects on photosynthesis, respiration and grain filling. The survival and the ability to set seed of *CcHyPRP*-transgenics under multiple stress conditions clearly indicate that the CcHyPRP confers protection to the plants at all stages of growth and development. *O. sativa* drought-induced *LTP* gene conferred drought tolerance at vegetative and reproductive stages in transgenic rice plants (Guo et al., 2013b). Overexpression of *Arabidopsis* Enhanced Drought Tolerance 1/HOMEODOMAIN GLABROUS11 gene in rice conferred increased drought stress tolerance and higher grain yield in transgenic rice plants under stress conditions (Yu et al., 2013).

Increased activities of catalase, SOD and lower levels of MDA were observed in *CcHyPRP*-transgenics as compared to the control plants (Figure 5). Higher levels of ROS produced during stress caused damage to the cellular macromolecules leading to MDA accumulation which inturn affected the stability of cell membranes (Gill and Tuteja, 2010). Transgenic rice and *Arabidopsis* expressing *Os*SUV3 dual helicase and *Cc*CDR showed lesser lipid peroxidation and increased activities of antioxidant enzymes (Tuteja et al., 2013; Tamirisa et al., 2014a). Transgenic rice plants, expressing *Cc*HyPRP, revealed increased chlorophyll content as compared to the control plants under different stress conditions. The amount of chlorophyll content is known to serve as an indicator for abiotic stress tolerance in plants (Willits and Peet, 2001; Li et al., 2006). The presence of higher chlorophyll content in transgenic rice plants subjected to stress conditions may be attributed to the maintenance of equilibrium of intracellular ROS levels. Transgenic *Arabidopsis* and tobacco plants expressing *Cc*CYP and *Cc*CDR were found to exhibit higher chlorophyll contents under different abiotic stress conditions owing to the efficient scavenging of ROS (Sekhar et al., 2010; Tamirisa et al., 2014b).

*CcHyPRP*-transgenic plants exhibited increased resistance to blast disease caused by *M. grisea* as evidenced by lesser number of lesions and their length (Figure 6). These results suggest that CcHyPRP participates in different signaling pathways which mediate responses to both biotic and abiotic factors. Upon overexpression of MYB96 protein, which is required for ABA-dependent SA biosynthesis, caused increased PR gene expression and enhanced resistance against pathogens (Seo and Park, 2010). Expression of OsNAC6 gene, a member of the NAC transcription factor gene family, in rice showed improved tolerance to dehydration, salt stress, and exhibited increased resistance to blast disease (Nakashima et al., 2007).

Higher endochitinase levels were found in CaMV35S-*CcHyPRP* rice plants when compared to untransformed control plants. Furthermore, RT-PCR analysis revealed higher levels of rice endochitinase gene transcripts in the transgenic rice plants (Figure 6). The higher endochitinase levels observed in the transgenics plausibly contribute to the enhanced tolerance against *M. grisea*. Chitinase catalyzes the hydrolysis of β-1, 4-linkage of the *N*-acetylglucosamine polymer of chitin, a major component of the fungal cell wall and inhibit the growth of fungal pathogens (Datta et al., 2001). Transgenic rice plants constitutively expressing either *Cht-2* or *Cht-3* genes showed significantly higher resistance against different *M. grisea* races (Nishizawa et al., 1999).

Enhanced levels of bZIP transcripts observed in transgenic plants as compared to the control plants, under identical conditions (Figure 7), indicate the involvement of *Cc*HyPRP in modulating the expression of bZIP, thereby contributing to increased tolerance/resistance to both abiotic and biotic stresses. Earlier, it was reported that bZIP transcription factors regulate various genes in response to abiotic stress and pathogen defense besides seed maturation and flower development (Jakoby et al., 2002; Alves et al., 2013). Overexpression of wheat bZIP transcription factor (*Ta*bZIP60) in *Arabidopsis* resulted in significantly improved tolerance to drought, salt, and freezing stresses (Zhang et al., 2014). In rice, the presence or absence of the qLTG3-1 gene, controlling low temperature tolerance at the seedling stage, revealed a major effect on the defense and other gene regulons (Fujino and Matsuda, 2010). Overexpression of *EARLI1* in *Arabidopsis* resulted in improved germination, root elongation and reduction of sodium accumulation in leaves under salt stress as well as the germinability under low-temperature stress (Xu et al., 2011).

The increased endochitinase levels found in transgenics are plausibly due to the involvement of *Cc*HyPRP as one of the components in the defense signaling cascade. In soybean, a HyPRP (*Gm*HyPRP) gene showed higher expression levels in response to the pathogenic fungus (*Phakopsora pachyrhizi)* which causes Asian soybean rust disease (Neto et al., 2013). Jung et al. (2009) reported the involvement of *A. thaliana* HyPRP (AZI1) in plant defense against the *Pseudomonas syringae*. Based on the functional validation of *Poncirus trifoliata HyPRP (Ptr*HyPRP), it was reported that *Ptr*HyPRP played an essential role for cold tolerance in transgenic plants (Peng et al., 2015). The main stress perception-to-signaling event seems to occur at the cell wall-plasma membrane interface where different proteins, such as arabinogalactans (AGPs), proline-rich proteins (PRPs), and receptor-like protein kinases, are present abundantly. Further, these proteins are involved in development, embryogenesis, cell-to-cell contacts and programmed cell death (Zagorchev et al., 2014). Since *Cc*HyPRP mainly localize at the cell wall, it might interact with the components of signaling cascade and participate in signal transduction. Hence, the overexpression of *CcHyPRP* gene resulted in upregulation of bZIP transcription factor and other stress related genes coding for enzymes responsible for the detoxification of ROS, culminating in multiple stress tolerance. However, further studies are needed to unravel the precise role of *Cc*HyPRP with other genes involved in biotic and abiotic stress tolerance.

To sum up, the present results amply suggest that the transgenic rice plants are able to withstand both biotic and abiotic stresses owing to the expression of *Cc*HyPRP protein which bestows resistance/tolerance to different stress conditions. Higher biomass, increased root, shoot lengths and grain number, under various stress conditions, demonstrate the superior performance of transgenic rice plants at both vegetative and reproductive stages. Higher levels of catalase and SOD activities are responsible for the elimination of ROS thus improving the cellular environment in the transgenic plants. Furthermore, activation of bZIP and chitinase genes also contributed for enhanced tolerance/resistance against abiotic and biotic stress conditions. The overall results indicate that the *Cc*HyPRP has an explicit functional role in conferring tolerance to drought, salt and heat stress conditions besides resistance to blast disease.

## Author contributions

Conceived and designed the experiments: VK, DV. Performed the experiments: SM, ST. Analyzed the data: VK, SM, ST. Wrote the paper: ST, SM, VK.

### Conflict of interest statement

The authors declare that the research was conducted in the absence of any commercial or financial relationships that could be construed as a potential conflict of interest.
