# Supplementary figures and images for: Pigeonpea Hybrid-Proline-Rich Protein (CcHyPRP) Confers Biotic and Abiotic Stress Tolerance in Transgenic Rice
Source: Front Plant Sci. 2016 Jan 22;6:1167. doi: 10.3389/fpls.2015.01167 (PMC4722794; doi:10.3389/fpls.2015.01167)

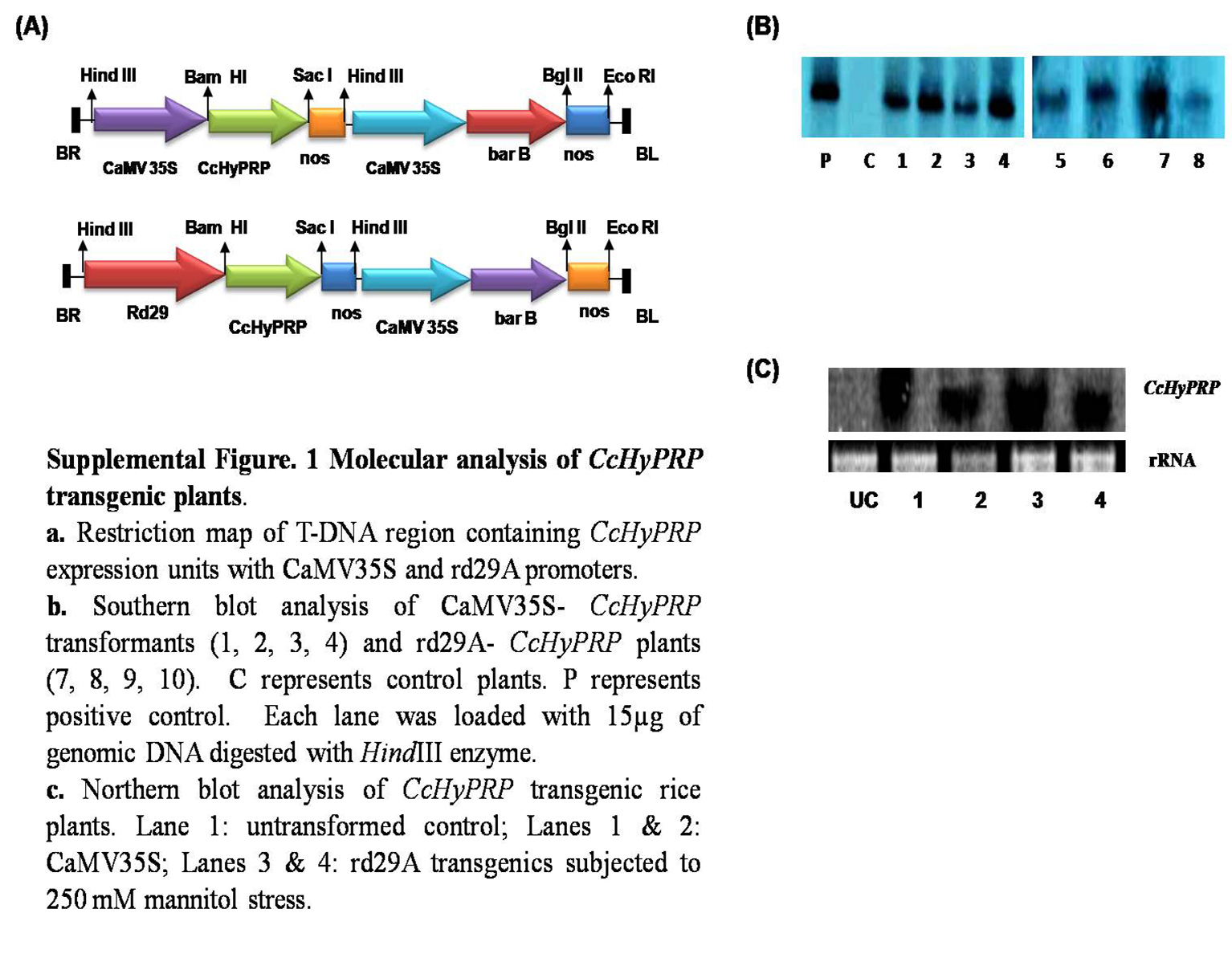

Supplement: Supplementary file 1 [file Image1.JPEG]

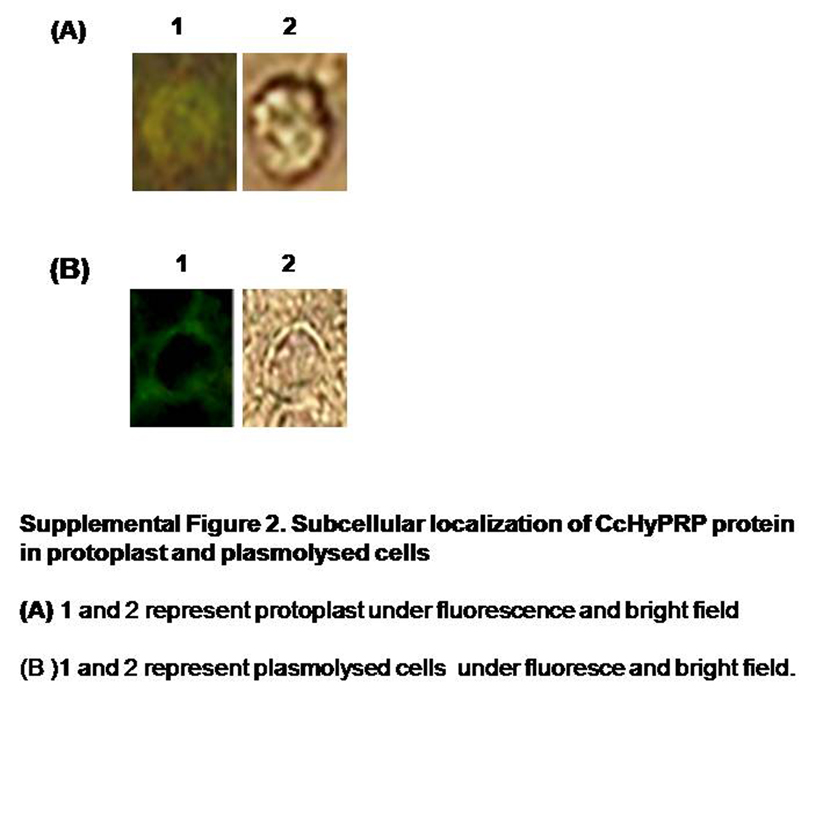

Supplement: Supplementary file 2 [file Image2.JPEG]

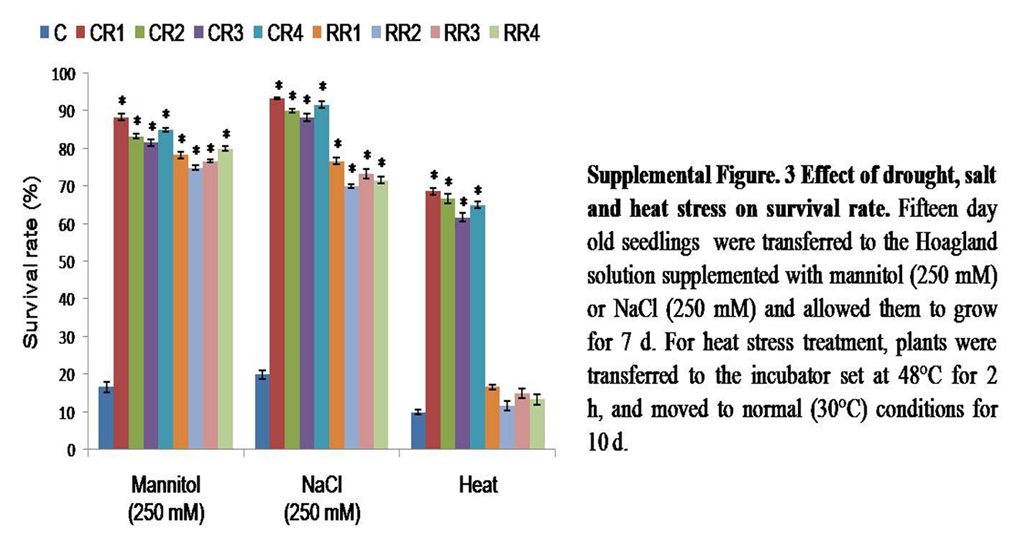

Supplement: Supplementary file 3 [file Image3.JPEG]

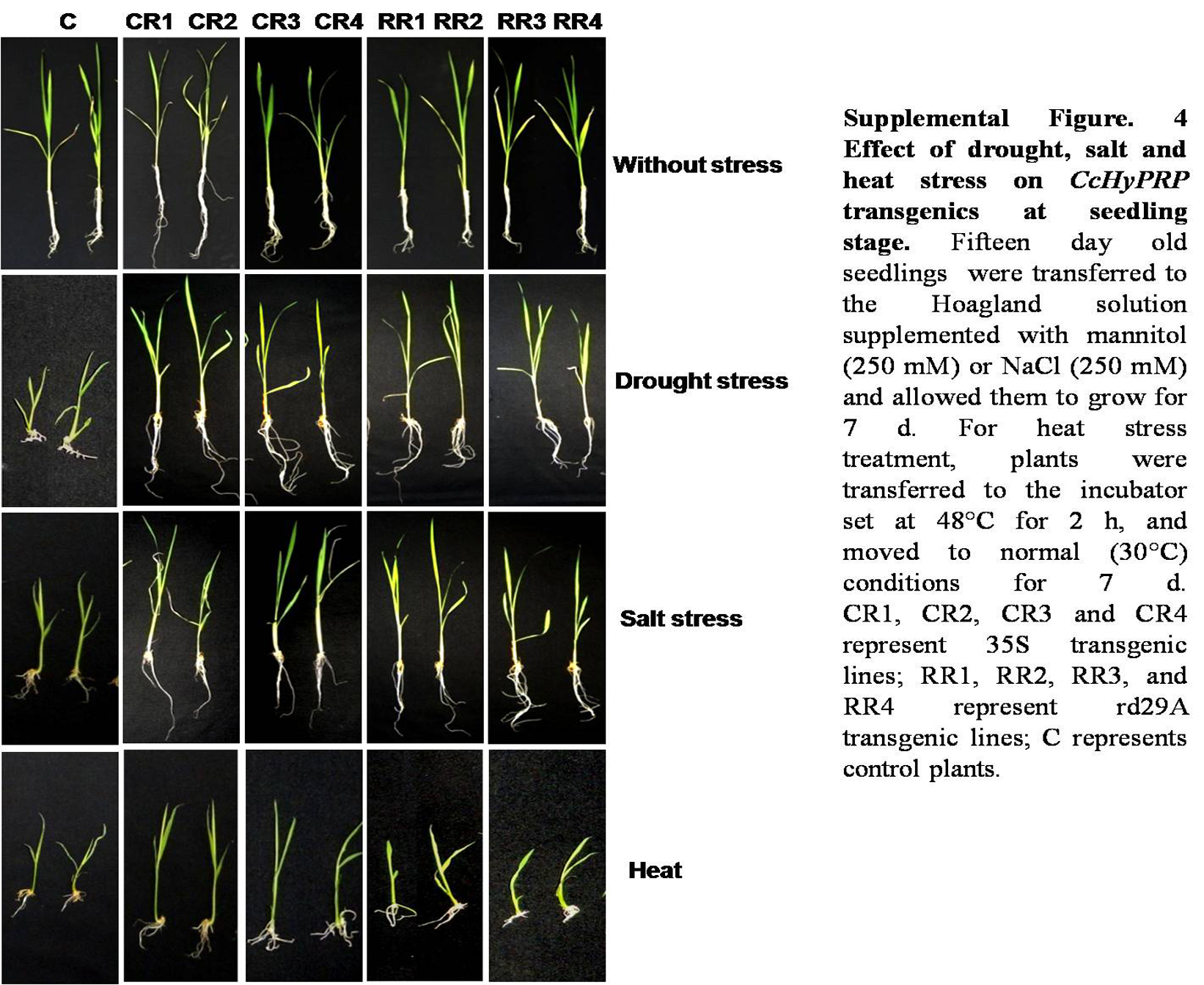

Supplement: Supplementary file 4 [file Image4.JPEG]
